# Supplementary material for: Rapid turnover of life-cycle-related genes in the brown algae
Source: Genome Biol. 2019 Feb 14;20:35. doi: 10.1186/s13059-019-1630-6 (PMC6374913; doi:10.1186/s13059-019-1630-6)
Supplement: Supplementary file 3 — Protein alignments, corresponding Gblocks html file and CDS sequences for the lineage conserved gametophyte-biased genes. (ZIP 2109 kb) [file 13059_2019_1630_MOESM3_ESM.zip › Con_GA_alignments/Ec-23_001600.aa_ali.fasta-gb.htm]

Ec-23\_001600.aa.fasta.trans.aa\_ali.fasta


## Gblocks 0.91b Results

Processed file: **Ec-23\_001600.aa.fasta.trans.aa\_ali.fasta**  
Number of sequences: **2**  
Alignment assumed to be: **Protein**  
New number of positions: **341** (selected positions are underlined in blue)

```
                         10        20        30        40        50        60
                 =========+=========+=========+=========+=========+=========+
Ec-23_001600     -------MNLELLDPFAKTVPDHVDSVLSYGVEDATVCCFGKRGAYLAVGYATGIVVIWD
MSTRG.24378.1    RVLVRETMNLELLDPFAKTVPDHVDSVLSYGVEDATVCCFDKRGAYLAVGYATGIVVIWD
                        #####################################################


                         70        80        90       100       110       120
                 =========+=========+=========+=========+=========+=========+
Ec-23_001600     FVTRGIGRVVEAHARAITALSWARGGRRLLSASHDQRLHYWALEPPRPELIRVICLDSPI
MSTRG.24378.1    FVTRGIGRVIEAHARGITALSWARGGRRLLSASHDQRLHYWALEPARPELIRVICLDSPI
                 ############################################################


                        130       140       150       160       170       180
                 =========+=========+=========+=========+=========+=========+
Ec-23_001600     MSALMLPGKYPRAMALTADGVAHVVDIKENTADAVSPAAATIISPPSAASSAGNPSPAKK
MSTRG.24378.1    MSALMLPGKYPRALALTADGVSHLVDIKENVTKAVCFTDRTIVSPPATASAAGNPSPAKK
                 ############################################################


                        190       200       210       220       230       240
                 =========+=========+=========+=========+=========+=========+
Ec-23_001600     PRPAGQAAAAATANRG-----PALPAVLCLALDRRASRVCLGTCQGQVLSVGTKDGDVSA
MSTRG.24378.1    ARSSSWTEQPAAPSNGSTSGASGMPAVLCLALDRRAPRVCLGTSSGQVLSVGTSNGEVSR
                 ################        ####################################


                        250       260       270       280       290       300
                 =========+=========+=========+=========+=========+=========+
Ec-23_001600     SMTVAGCAKVKQMDLSRDGKWMLVNGGERVIRMLERESTTGRSVRDFQDVVNRTRWQCCR
MSTRG.24378.1    SMAVVGCSKVKQLDLSRDGKWMLVNAGEKVIRMLERESTTGRSVRDFQDVVNRTRWQCCR
                 ############################################################


                        310       320       330       340       350       360
                 =========+=========+=========+=========+=========+=========+
Ec-23_001600     FTGDSEYIAGGSRAEGTCSIYLWTIEGHLVTRLEGPNLGLLSLTRHPVRPFLAAALTNGK
MSTRG.24378.1    FTGDSEYIAGGSRAEAACSIYIWTVEGHLVTRLDGPNLGLLHLMRHPVRPFLAAAL----
                 ########################################################    


                        370       380       390       400       410       420
                 =========+=========+=========+=========+=========+=========+
Ec-23_001600     VQLWGTTPIANWTAFAADFEELEENVEYVEKEDEFDVVVEEGDPEGTKRKAEKEKQQEEE
MSTRG.24378.1    ------------------------------------------------------------
                                                                             


                        430       440       450       460       470       480
                 =========+=========+=========+=========+=========+=========+
Ec-23_001600     AVVSIDAIDTVDAWSTDSEGEDRLQPFYLSTEPGAAAREGWYDLDVQTSKRGISSKPGGG
MSTRG.24378.1    ------------------------------------------------------------
                                                                             


                        490       500       510       520       530       540
                 =========+=========+=========+=========+=========+=========+
Ec-23_001600     RWEDKEEWTSVRTGDGGGVKKGGGGAKKKKPSNREFGKRGKSHAGKNNNQAPSSKARAAT
MSTRG.24378.1    ------------------------------------------------------------
                                                                             


                        550       560
                 =========+=========+
Ec-23_001600     DAPSGSGRGGGRRRRRRQRR
MSTRG.24378.1    --------------------
```

```
Parameters used
Minimum Number Of Sequences For A Conserved Position: 2
Minimum Number Of Sequences For A Flanking Position: 2
Maximum Number Of Contiguous Nonconserved Positions: 8
Minimum Length Of A Block: 10
Allowed Gap Positions: None
Use Similarity Matrices: Yes
```

```
Flank positions of the 2 selected block(s)
Flanks: [8  196]  [205  356]  

New number of positions in Ec-23_001600.aa.fasta.trans.aa_ali.fasta-gb:  341  (60% of the original 560 positions)
```
